# Placental programming of blood pressure in Indian children

**DOI:** 10.1111/j.1651-2227.2010.02102.x

**Published:** 2011-05

**Authors:** Nicola R Winder, Ghattu V Krishnaveni, Jacqueline C Hill, Chitra LS Karat, Caroline HD Fall, Sargoor R Veena, David JP Barker

**Affiliations:** 1 MRC Lifecourse Epidemiology Unit, Southampton General Hospital, University of SouthamptonSouthampton, UK; 2 Epidemiology Research Unit, CSI Holdsworth Memorial HospitalMysore 570021, India

**Keywords:** Birth size, Blood pressure, Indian children, Maternal height, Placental surface

## Abstract

**Aim:**

To determine whether the size and shape of the placental surface predict blood pressure in childhood.

**Methods:**

We studied blood pressure in 471 nine-year-old Indian children whose placental length, breadth and weight were measured in a prospective birth cohort study.

**Results:**

In the daughters of short mothers (<median height), systolic blood pressure (SBP) rose as placental breadth increased (β = 0.69 mmHg/cm, p = 0.05) and as the ratio of placental surface area to birthweight increased (p = 0.0003). In the daughters of tall mothers, SBP rose as the difference between placental length and breadth increased (β = 1.40 mmHg/cm, p = 0.007), that is as the surface became more oval. Among boys, associations with placental size were only statistically significant after adjusting for current BMI and height. After adjustment, SBP rose as placental breadth, area and weight decreased (for breadth β = −0.68 mmHg/cm, p < 0.05 for all three measurements).

**Conclusions:**

The size and shape of the placental surface predict childhood blood pressure. Blood pressure may be programmed by variation in the normal processes of placentation: these include implantation, expansion of the chorionic surface in mid-gestation and compensatory expansion of the chorionic surface in late gestation.

## Introduction

People whose birthweights were towards the lower end of the normal range have higher blood pressures and are at increased risk of developing hypertension in later life (1–3). This is thought to reflect foetal programming, the process by which malnutrition and other adverse influences during development alter gene expression and programme the body’s structures and function for life (4,5). These adverse influences may also slow foetal growth, leading to low birthweight (6). A baby’s birthweight reflects its success in obtaining nutrients from its mother. The source of these nutrients is not only the mother’s current diet but her metabolism, which is a product of her lifetime’s nutrition (7). Height is one indicator of a woman’s nutrition in early life (8), and women who are short have lower rates of protein synthesis during pregnancy than do women who are tall (9).

A baby’s birthweight also depends on the placenta’s ability to transport nutrients to it from its mother (6). Small babies generally have small placentas (10), which suggests that placental size is an indicator of placental function. In some circumstances, however, an undernourished baby can expand its placental surface to extract more nutrients from the mother (11). This leads to high placental weight in relation to birthweight. Both low placental weight and high placental weight in relation to birthweight have been shown to predict later hypertension (12–14). Placental weight has inconsistent associations with blood pressure levels in children. There are reported associations between increased levels of blood pressure and low placental weight and a high ratio of placental weight to birthweight (15,16). Other studies have found no associations (17). While the size of the placenta is linked to foetal nutrition, which nutrients are delivered to the foetus is conditional on their availability in the maternal circulation. This will be related to the mother’s body size. The effects of placental size on later hypertension are conditioned by the mother’s body size (11) because her body is the source of nutrients.

The weight of the placenta does not distinguish its thickness from its surface area. To increase the surface for nutrient and oxygen exchange, the placenta can expand its invasion across the surface of the uterine lining or invade the maternal spiral arteries more deeply. The long-term consequences of these may be different.

The surface of the placenta is generally described as oval or round. To measure the ovality, two so-called diameters of the surface were routinely recorded in some hospitals (11). The maximal diameter described the length of the surface, while the lesser one bisecting it at right angles described the breadth. Studies in Finland and Holland have shown that hypertension in the offspring in later life is related to the size of the placental surface (11). This relation depends more on the breadth of the surface than on its length.

We measured the length and breadth of the placental surface in a study of newborn babies in Mysore, South India (18). The children’s blood pressures were measured at the age of 9 years. We hypothesized that blood pressure levels would be related to the size of the placental surface and would relate more to the breadth than the length. We also hypothesized that the associations would depend on the mother’s height. The relation between placental size and later hypertension differs in the two sexes (19). We therefore examined boys and girls separately.

## Methods

In 1997, the Mysore Parthenon study recruited pregnant women attending the antenatal clinic at the Holdsworth Memorial Hospital (HMH) in Mysore, South India (18,20). The hospital ethical committee approved the study, and informed written consent was obtained from the parents and children. All women who had a singleton pregnancy and who were not diabetic before pregnancy were eligible. Out of 1233 eligible women, 830 (67%) took part in the study. At 30 ± 2 weeks of gestation, their body size was measured, including their height, using standardized methods. One hundred and fifty-six women delivered elsewhere and were not followed up any further: the remaining 674 women delivered live-born babies at HMH. Seven babies were stillborn and four had major congenital abnormalities. Neonatal anthropometric measurements were made on the remaining 663 within 72 h of birth by one of the four trained measurers, again using standardized methods. Weight was measured using digital weighing scales (Seca, Germany); crown-heel length was measured using a Harpenden neonatal stadiometer (CMS instruments, London, UK); head circumference was measured with blank anthropometric tape, marked and measured against a fixed ruler.

Placental dimensions were measured in 653 of the babies. The placentae were initially checked for completeness, and the cord clamp was released to allow the blood to drain. The amnion was stripped off, and the chorion was trimmed close to the placental edge. The cord was cut flush with the placenta, and any obvious clots were removed. The placenta was weighed using an electronic weighing machine. It was then placed on a flat surface, with the cotyledons facing upwards. The longest diameter (length) was identified by eye and measured using a graduated transparent plastic ruler placed on the surface. The diameter perpendicular to the length was defined as the breadth and was measured in the same way.

### Postnatal follow-up

**Figure 1:**
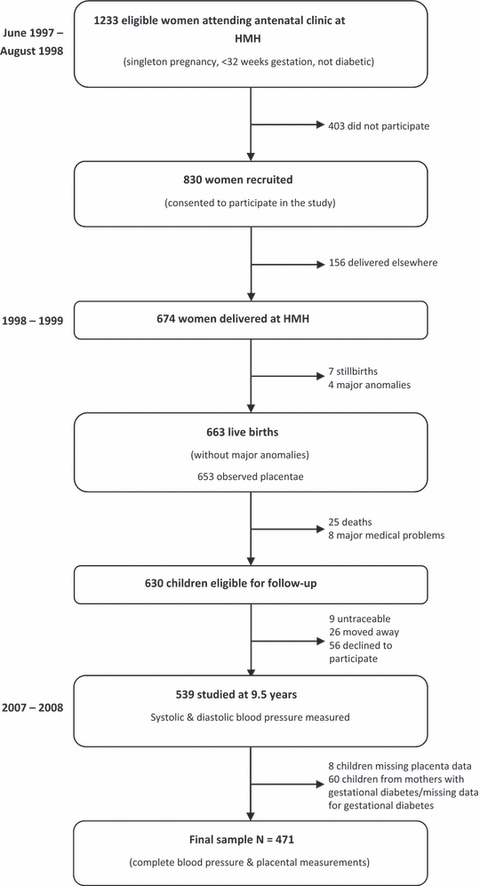
Flow diagram of study participants.

The children were followed up annually from birth until 5 years of age, and every six months thereafter. Eight children were excluded because of major medical conditions, while a further 25 children died. Ninety-one children did not attend the nine and a half year follow-up (nine untraceable, 26 moved away and 56 declined to participate) so that 539 children were examined (Figure 1). Further anthropometry was carried out including weight (Salter, Tonbridge, Kent, UK), height (Microtoise; CMS instruments) and mid-upper arm circumference. Systolic and diastolic blood pressures were measured in the left arm using an automated blood pressure monitor (Dinamap8100, Criticon, FL, USA). Appropriate-sized cuffs were selected for use based on the mid-upper arm circumference. Two recordings were made after five minutes seated at rest, and the average taken. The observers were unaware of the neonatal measurements of the children. The techniques used by different observers were standardized by regular intra- and inter-observer variation studies.

### Analysis sample

Blood pressure was recorded for all 539 children who attended the 9.5-year follow-up. Because placental size is increased in gestational diabetes, we excluded from the analysis 35 children whose mothers developed diabetes during gestation and 25 children for whom maternal glucose tolerance test data were missing. We included seven children born to mothers who developed hypertension and 29 babies born preterm. Placental measurements were missing for eight children, and hence our study sample comprises the remaining 471 children with complete measurements of the placenta.

### Statistical methods

The data included the weight of the placenta and the length and breadth of its surface. Placental area was calculated assuming an elliptical surface, using the maximal diameter (length) × lesser diameter (breadth) × π/4 (11). We calculated the difference between the length and breadth of the surface and the ratio of placental area to birthweight. We examined associations between placental variables and blood pressure using linear regression, unadjusted and then adjusted for the child’s body mass index (BMI) and height at 9.5 years; where p values from adjusted models are used, this is stated. We used linear regression to examine interactions between the effects on blood pressure of placental size and maternal height. Among girls, there were interactions between the effects of placental size and maternal height (see Results); and, as in the analyses of the Helsinki Birth Cohort (11,19), we examined separate models for girls born to mothers above and below the median height (154.5 cm). There were no similar interactions between placental size and maternal height in boys.

## Results

Table 1 shows the mean measurements of the 471 mothers, placentas, babies and children. None of the mothers smoked tobacco. At birth, boys were larger than girls, but their placental size was similar. Twelve boys and eight girls had systolic hypertension, using standard criteria (21).

**Table 1:** Characteristics of the mothers, placentas, babies and children in the Mysore Parthenon cohort

	n	Mean/[%]	(SD)				
Mother							
Age (years)	471	23.5	(4.0)				
Height (cm) at 30 weeks gestation	471	154.3	(5.2)				
Weight (kg) at 30 weeks gestation	471	55.8	(8.7)				
Body mass index (BMI) (kg/m^2^) at 30 weeks gestation	471	23.4	(3.5)				
Parity							
0	241	[51.2]					
1	151	[32.0]					
2 or more	79	[16.8]					

	Boys			Girls			
			
	n	Mean	(SD)	n	Mean	(SD)	p-value
Placenta							
Length (cm)	228	19.6	(2.1)	243	19.4	(2.0)	0.2
Breadth (cm)	228	17.0	(1.8)	243	17.0	(1.8)	0.995
Weight (g)	228	410	(81)	243	407	(85)	0.7
Length–breadth (cm)	228	2.6	(1.8)	243	2.4	(1.5)	0.08
Area (cm^2^)	228	263.5	(50.1)	243	259.9	(48.2)	0.4
Baby							
Gestational age (weeks)	228	39.2	(1.6)	243	39.4	(1.5)	0.2
Weight (g)	228	2898	(467)	243	2804	(387)	0.02
Crown-heel length (cm)	228	48.8	(2.3)	243	48.2	(2.1)	0.004
Head circumference (cm)	228	34.1	(1.4)	243	33.5	(1.2)	<0.0001
Children at 9.5 years							
Age (years)	228	9.4	(0.1)	243	9.4	(0.1)	0.29
Systolic B.P (mmHg)	228	102.0	(8.3)	243	99.9	(8.5)	0.008
Diastolic B.P (mmHg)	228	58.5	(6.9)	243	58.0	(6.6)	0.3
Height (cm)	228	131.3	(5.5)	243	130.2	(5.9)	0.05
Weight (kg)	228	25.3	(4.4)	243	24.8	(4.7)	0.3
BMI (kg/m^2^)	228	14.6	(1.8)	243	14.5	(1.9)	0.8

p values for the differences between boys and girls using unpaired *t*-tests.

There were no significant differences in birthweight or any of the placental measurements between the 471 children included in the study sample and the 118 children who were eligible but who were not included, either because of incomplete placental measurements or because of loss to follow-up (birthweight: 2850 g vs. 2781 g, p = 0.2; placental length: 19.5 cm vs. 19.3 cm p = 0.4; placental breadth: 17.0 cm vs. 16.7 cm p = 0.1; placental area: 261.6 cm vs. 255.1 cm p = 0.2; placental weight: 0.408 kg vs. 0.411 kg p = 0.8). However, there was a significant difference in maternal height between these two groups: 154.3 cm vs. 156.4 cm, p = 0.0002).

Among boys, neither systolic nor diastolic pressure was related to birthweight, head circumference, birth length or the length of gestation. Table 2 shows the trends in blood pressure with placental size. Systolic pressure tended to rise as placental breadth, weight and area decreased. These trends became statistically significant after adjusting for the child’s current BMI and height (for breadth β = −0.68 mmHg/cm). The trends with breadth and weight also became statistically significant after adjusting for birth weight (p = 0.04 for breadth and p = 0.03 for weight). Systolic pressure tended to rise as the ratio of placental area to birth weight decreased; however, this trend was not statistically significant. Diastolic pressure was unrelated to placental size.

**Table 2:** Blood pressure at 9.5 years among boys and girls according to placental size

	Boys						Girls					
		
	Systolic blood pressure (mmHg)			Diastolic blood pressure (mmHg)			Systolic blood pressure (mmHg)			Diastolic blood pressure (mmHg)		
				
Placenta	n	Mean	(SD)	n	Mean	(SD)	n	Mean	(SD)	n	Mean	(SD)
Breadth (cm)												
≤16	68	102.9	(8.9)	68	58.7	(6.5)	83	100.6	(8.4)	83	59.2	(6.3)
17	53	102.6	(7.8)	53	58.9	(7.7)	50	98.4	(8.1)	50	56.1	(7.2)
18	46	100.3	(8.5)	46	58.6	(6.4)	49	98.5	(9.0)	49	58.3	(6.7)
>18	61	101.6	(8.0)	61	58.0	(7.0)	61	101.4	(8.5)	61	57.5	(6.2)
p for trend (unadjusted)	228	0.09		228	0.67		243	0.50		243	0.12	
p for trend*	228	0.01		228	0.50		243	0.83		243	0.09	
Length (cm)												
≤18	56	101.8	(8.4)	56	58.3	(7.1)	64	99.1	(8.2)	64	58.5	(5.8)
19.5	51	103.2	(8.1)	51	59.4	(5.9)	68	99.8	(8.6)	68	58.7	(8.0)
21	67	101.5	(8.1)	67	58.7	(7.2)	64	99.7	(9.1)	64	56.9	(5.9)
>21	54	101.6	(8.9)	54	57.8	(7.1)	47	101.4	(8.3)	47	57.6	(6.4)
p for trend (unadjusted)	228	0.60		228	0.75		243	0.07		243	0.29	
p for trend*	228	0.15		228	0.53		243	0.59		243	0.19	
Weight (g)												
≤350	56	102.5	(8.9)	56	58.5	(7.6)	58	99.4	(8.1)	58	58.6	(7.4)
400	57	101.1	(7.6)	57	58.7	(6.5)	80	99.6	(8.5)	80	58.6	(6.5)
450	58	103.6	(8.5)	58	59.4	(6.6)	44	100.1	(9.3)	44	57.9	(5.6)
>450	57	100.6	(8.2)	57	57.5	(6.8)	61	100.6	(8.6)	61	56.6	(6.6)
p for trend (unadjusted)	228	0.15		228	0.24		243	0.80		243	0.02	
p for trend*	228	0.01		228	0.12		243	0.24		243	0.01	
Area (cm^2^)												
≤225	50	102.4	(8.3)	50	59.2	(7.5)	59	99.7	(8.3)	59	58.9	(5.8)
260	54	102.8	(9.0)	54	58.3	(6.1)	64	99.3	(8.6)	64	58.9	(7.7)
300	74	102.1	(7.6)	74	58.8	(7.1)	67	99.4	(8.7)	67	56.5	(6.3)
>300	50	100.5	(8.6)	50	57.7	(6.8)	53	101.5	(8.5)	53	57.6	(6.2)
p for trend (unadjusted)	228	0.22		228	0.70		243	0.18		243	0.14	
p for trend*	228	0.03		228	0.49		243	0.86		243	0.10	
Area/birth weight (cm^2^/kg)												
≤80	62	102.9	(8.9)	62	59.6	(7.5)	49	99.2	(8.5)	49	58.4	(5.6)
90	51	102.6	(8.4)	51	57.9	(7.3)	50	100.2	(7.0)	50	58.3	(6.5)
100	52	101.9	(7.9)	52	58.2	(6.9)	65	98.2	(9.3)	65	57.4	(7.6)
>100	63	100.6	(8.0)	63	58.3	(5.8)	79	101.6	(8.6)	79	57.9	(6.4)
p for trend (unadjusted)	228	0.10		228	0.22		243	0.01		243	0.93	
p for trend*	228	0.18		228	0.30		243	0.004		243	0.89	

The means are unadjusted.

p values were obtained by linear regression using all variables as continuous.

* p values adjusted for the child’s current body mass index and height.

Among girls, systolic and diastolic pressure fell as birthweight increased (systolic pressure fell by 4.2 mmHg per kg increase in birthweight, 95% CI 1.4–6.9, p = 0.003; diastolic pressure fell by 2.6 mmHg per kg increase, 95% CI 0.3–4.9, p = 0.03, after adjusting for current BMI). Systolic pressure in girls also fell as birth length increased (p = 0.04, adjusted for current BMI). It was not related to head circumference or to the length of gestation. Table 2 shows the trends in blood pressure with placental size. Systolic blood pressure rose as the ratio of area to birthweight increased. Diastolic blood pressure fell as placental weight increased. In Table 3, the trends in blood pressure with placental size among girls are divided according to the mother’s median height. Among girls whose mothers’ height was below the median, systolic pressure rose as placental breadth increased (β = 0.69 mmHg/cm) and as the ratio of placental area to birthweight increased. In a simultaneous regression, both larger placental area and lower birthweight were associated with higher systolic pressure (p = 0.001 and 0.002, respectively). Diastolic pressure also rose as the ratio of placental area to birthweight increased; however, this was a weaker trend than that for systolic pressure.

**Table 3:** Blood pressure at 9.5 years among girls according to mother’s height and placental size

	Short mothers						Tall mothers					
		
	Systolic blood pressure (mmHg)			Diastolic blood pressure (mmHg)			Systolic blood pressure (mmHg)			Diastolic blood pressure (mmHg)		
				
Placenta	n	Mean	(SD)	n	Mean	(SD)	n	Mean	(SD)	n	Mean	(SD)
Breadth (cm)												
≤16	49	98.8	(7.9)	49	58.7	(6.5)	34	103.2	(8.7)	34	60.0	(5.9)
17	25	98.1	(8.4)	25	56.7	(7.0)	25	98.7	(7.9)	25	55.4	(7.5)
18	22	100.6	(8.6)	22	59.2	(7.8)	27	96.8	(9.0)	27	57.4	(5.6)
>18	32	101.7	(9.2)	32	57.8	(6.2)	29	101.1	(7.8)	29	57.3	(6.4)
p for trend (unadjusted)	128	0.049		128	0.91		115	0.25		115	0.03	
p for trend*	128	0.11		128	0.83		115	0.09		115	0.02	
Length (cm)												
≤18	39	99.0	(7.6)	39	58.5	(5.9)	25	99.3	(9.1)	25	58.5	(5.7)
19.5	36	99.0	(9.4)	36	58.6	(8.8)	32	100.8	(7.6)	32	58.9	(7.1)
21	27	100.4	(8.3)	27	56.0	(5.2)	37	99.2	(9.7)	37	57.5	(6.3)
>21	26	100.9	(8.7)	26	59.3	(5.8)	21	102.00	(7.8)	21	55.4	(6.5)
p for trend (unadjusted)	128	0.17		128	0.90		115	0.24		115	0.07	
p for trend*	128	0.37		128	0.58		115	0.83		115	0.02	
Weight (g)												
≤350	34	100.9	(9.0)	34	59.1	(8.4)	24	97.3	(6.2)	24	57.9	(5.8)
400	51	99.5	(8.2)	51	59.1	(6.1)	29	99.9	(9.2)	29	57.6	(7.0)
450	17	97.0	(6.8)	17	54.8	(4.9)	27	102.1	(10.2)	27	59.8	(5.2)
>450	26	100.3	(9.4)	26	57.3	(6.0)	35	100.9	(8.2)	35	56.1	(7.1)
p for trend (unadjusted)	128	0.54		128	0.05		115	0.34		115	0.24	
p for trend*	128	0.23		128	0.10		115	0.92		115	0.09	
Area (cm^2^)												
≤225	36	98.6	(7.5)	36	58.3	(6.0)	23	101.6	(9.3)	23	59.7	(5.6)
260	35	98.7	(9.3)	35	58.7	(8.1)	29	100.1	(7.8)	29	59.2	(7.3)
300	33	99.9	(8.0)	33	56.8	(7.1)	34	98.8	(9.4)	34	56.2	(5.5)
>300	24	102.4	(9.1)	24	59.0	(5.2)	29	100.8	(8.2)	29	56.5	(6.9)
p for trend (unadjusted)	128	0.07		128	0.99		115	0.97		115	0.03	
p for trend*	128	0.18		128	0.70		115	0.40		115	0.01	
Area/birth weight (cm^2^/g)												
≤ 80	23	97.2	(6.8)	23	57.1	(4.7)	26	100.9	(9.6)	26	59.6	(6.2)
90	26	99.3	(7.0)	26	58.4	(6.5)	24	101.0	(7.0)	24	58.1	(6.7)
100	39	98.9	(9.4)	39	57.9	(8.1)	26	97.1	(9.1)	26	56.7	(7.0)
>100	40	102.0	(9.0)	40	58.8	(6.7)	39	101.2	(8.4)	39	56.9	(6.1)
p for trend (unadjusted)	128	0.0003		128	0.06		115	0.83		115	0.07	
p for trend*	128	0.0002		128	0.0499		115	0.68		115	0.08	

The means are unadjusted.

p values were obtained by linear regression using all variables as continuous.

* p values adjusted for the child’s current body mass index and height.

Among girls whose mothers’ height was above the median, there were no trends in systolic blood pressure with either placental length, breadth or area (Table 3), but systolic pressure rose as the difference between the diameters increased (β = 1.40 mmHg/cm, p = 0.007), that is as the placental surface became more oval. In contrast to systolic pressure, diastolic pressure rose as placental breadth, length and area decreased, but was unrelated to the difference between diameters. The differing trends in systolic pressure with placental size in the two maternal height groups were statistically significant (p for interaction = 0.03 for breadth, 0.02 for area/birthweight, and 0.03 for the difference between length and breadth).

## Discussion

We found that the blood pressures of 9-year-old children in south India were related to the size and shape of the placental surface at birth. We suggest that these associations reflect the role of placental function in programming blood pressure (22). Consistent with findings in the Helsinki birth Cohort, we found different associations in boys and girls (19). Boys grow faster than girls from an early stage of gestation, even from before implantation, and this makes them more vulnerable if their nutrition is compromised (8,23). More newborn boys than girls have retarded growth and placental abnormalities, and most of them die during the perinatal period (24). The associations between blood pressure and placental size were modified by adjustment for the child’s current body size. This reflects the known association between raised blood pressure and rapid postnatal growth (3).

We found that three different placental phenotypes predicted raised blood pressure depending on the child’s sex and the mother’s height. Common to each phenotype was that blood pressure was related to the shape or size of the placental surface and to the breadth rather than to the length. Many studies have shown that raised blood pressure in children and adults is related to lower birthweight within the normal range (1–3,15–17). This suggests that blood pressure levels are linked to foetal malnutrition (6). We now examine each of the three placental phenotypes and discuss their possible relation to foetal malnutrition.

### Boys, small placental surface

In boys, higher systolic blood pressure was associated with smaller placental area. This association depended on reduction in placental breadth rather than length. This is consistent with findings among men in Holland, in whom hypertension was related to a short placental breadth but not to length (25). One suggestion is that tissue along the breadth is more closely related to nutrient delivery to the foetus than tissue along the length of the surface, and shorter breadth results in lesser delivery (26). The length and breadth of the placental surface are established by growth of the chorionic surface in mid-gestation. We suggest that reduced growth in placental breadth is associated with foetal malnutrition and raised blood pressure in boys.

Among boys, the effects of placental size on blood pressure were not conditioned by the mothers’ height. In the Helsinki Birth Cohort (19), the effects of placental surface area on hypertension were not related to the mother’s height in men, but were stronger among women with short mothers. This led to the suggestion that, compared to boys, the nutrition of girls *in utero* depends more on the mothers’ lifetime nutrition and metabolism, reflected in her height than on the mothers’ diet in pregnancy (19).

### Girls, large placental surface

In girls whose mother’s height was below the median, raised systolic pressure was associated with a large placental area in relation to birthweight. This association is therefore opposite to that seen in the Helsinki cohort, in which hypertension was associated with smaller placental area, especially in women with shorter mothers (19). Again, the association in our study depended more on the breadth of the placental surface than on its length. A study of men and women born in a maternity hospital in Preston, UK, showed that high placental weight in relation to birthweight was associated with later hypertension in the offspring (13). Measurements of the placental surface were not available. This observation has been replicated, and high placental weight in relation to birthweight has also been shown to predict coronary heart disease (14,27). In response to maternal undernutrition in mid-gestation, foetal lambs are able to extend the area of the placenta by expanding the individual cotyledons (28). This increases the area available for nutrient and oxygen exchange and, if normal nutrition is restored in late gestation, there is a larger lamb than there would otherwise have been. This is profitable for the farmer, and manipulation of placental size by changing the pasture of pregnant ewes is standard practice in sheep farming. There is evidence that a similar process occurs in humans and involves broadening of the placental surface rather than elongation (11,19). Compensatory placental expansion may be beneficial in some circumstances, but if the compensation is inadequate, and the foetus continues to be undernourished, the need to share its nutrients with an enlarged placenta may become an added metabolic burden that has long-term costs, which includes raised blood pressure.

### Girls, oval placental surface

In girls whose mothers’ height was above the median higher, systolic pressure was predicted by a larger difference between the breadth and length of the placental surface that is by a more oval-shaped surface. In contrast, diastolic pressure was not predicted by the shape of the surface but by its size, pressures rising with decreasing placental area. In pregnancies complicated by pre-eclampsia, a disorder that is initiated by impaired implantation, the placental surface is oval (26). We therefore suggest that the association between systolic pressure and an oval placenta reflects disruption of the processes of implantation, which includes spiral artery invasion and recruitment, with consequent foetal malnutrition. The association between small placental area and diastolic pressure may reflect reduced expansion of the chorionic surface in mid-gestation. We can offer no explanation as to why impaired implantation would affect systolic pressure while reduced expansion of the chorionic surface would affect diastolic pressure.

### Strengths and limitations of the study

In the Parthenon study, placental and newborn size and childhood blood pressure were measured by trained research staff according to standard protocols. None of the mothers smoked, and only seven had pregnancy-induced hypertension. The study has achieved high follow-up rates in the children; 80% of the original live-born babies of nondiabetic mothers and 84% of surviving children were studied at 9 years. The study is based on births in one hospital in Mysore, and the participants may therefore be unrepresentative of the whole Mysore population. At the time of the study, the Holdsworth Memorial Hospital was one of three large maternity units in Mysore. It is situated in a relatively poor area of the city, and most of the patients come from ‘lower middle-class and middle-class’ families. However, it is not a specialist referral hospital, and most women choose to deliver there because of its proximity to home. We do not think loss to follow-up would have introduced significant bias. Although the mothers of the children included in our study sample were significantly shorter than those lost to follow-up, differences in birthweight and placental measurements between these groups were small.

## Conclusion

We suggest that variations in three normal processes of placentation lead to foetal undernutrition and consequent raised blood pressure. The processes are those that accompany implantation and expansion of the chorionic surface in mid-gestation and compensatory expansion of the chorionic surface in late gestation. In girls, the effects of these disruptions on blood pressure are conditioned by the mother’s early nutrition as indicated by her height.

## References

[1] Law CM, de SM, Osmond C, Fayers PM, Barker DJ, Cruddas AM (1993). Initiation of hypertension *in utero* and its amplification throughout life. Br Med J.

[2] Adair L, Dahly D (2005). Developmental determinants of blood pressure in adults. Annu Rev Nutr.

[3] Eriksson JG, Forsen TJ, Kajantie E, Osmond C, Barker DJP (2007). Childhood growth and hypertension in later life. Hypertension.

[4] Barker DJP (1995). Fetal origins of coronary heart disease. Br Med J.

[5] Barker DJP (1998). Mothers, babies and disease in later life.

[6] Harding JE (2001). The nutritional basis of the fetal origins of adult disease. Int J Epidemiol.

[7] Jackson AA (2000). All that glitters. Br Nutr Foundation Annual Lecture. Nutr Bull.

[8] Tanner JM (1989). Fetus into man.

[9] Duggleby SL, Jackson AA (2001). Relationship of maternal protein turnover and lean body mass during pregnancy and birth length. Clin Sci.

[10] Sibley CP, Boyd RDH, Polin RA, Fox WW (1992). Mechanisms of transfer across the human placenta. Fetal and neonatal physiology.

[11] Barker DJP, Thornburg KL, Osmond C, Kajantie E, Eriksson JG (2010). The surface area of the placenta and hypertension in the offspring in later life. Int J Dev Biol.

[12] Barker DJP, Bull AR, Osmond C, Simmonds SJ (1990). Fetal and placental size and risk of hypertension in adult life. Br Med J.

[13] Campbell DM, Hall MH, Barker DJ, Cross J, Shiell AW, Godfrey KM (1996). Diet in pregnancy and the offspring’s blood pressure 40 years later. Br J Obstet Gynaecol.

[14] Eriksson J, Forsen T, Tuomilehto J, Osmond C, Barker D (2000). Fetal and childhood growth and hypertension in adult life. Hypertension.

[15] Moore VM, Miller AG, Boulton TJ, Cockington RA, Craug IH, Magarey AM (1996). Placental weight, birth measurements, and blood pressure at age 8 years. Arch Dis Child.

[16] Taylor SJC, Whincup PH, Cook DG, Papacosta O, Walker M (1997). Size at birth and blood pressure; cross sectional study in 8–11 year old children. Br Med J.

[17] Whincup P, Cook D, Papacosta O, Walker M (1995). Birth weight and blood pressure: cross sectional and longitudinal relations in childhood. Br Med J.

[18] Hill JC, Krishnaveni GV, Annamma I, Leary SD, Fall CHD (2005). Glucose tolerance in pregnancy in South India: relationships to neonatal anthropometry. Acta Obstet Gynecol Scand.

[19] Eriksson JG, Kajantie E, Osmond C, Thornburg K, Barker DJP (2010). Boys live dangerously in the womb. Am J Hum Biol.

[20] Krishnaveni GV, Veena SR, Hill JC, Kehoe S, Karat SC, Fall CHD (2010). Intrauterine exposure to maternal diabetes is associated with higher adiposity and insulin resistance and clustering of cardiovascular risk markers in Indian children. Diabetes Care.

[21] National High Blood Pressure Educational Program Working Group on High Blood Pressure and Adolescents (2004). The fourth report on the diagnosis, evaluation and treatment of high blood pressure in children and adolescents. Pediatrics.

[22] Jansson T, Powell TL (2007). Role of the placenta in fetal programming: underlying mechanisms and potential interventional approaches. Clin Sci.

[23] Pedersen JF (1980). Ultrasound evidence of sexual differences in fetal size in first trimester. Br Med J.

[24] Di Renzo GC, Rosati A, Sarti RD, Cruciani L, Cutuli AM (2007). Does fetal sex affect pregnancy outcome?. Gend Med.

[25] Barker DJP, Kajantie E, Eriksson JG, Alwasel SH, Fall CHD, Roseboom TJ, Burton GJ, Barker DJP, Moffett A, Thornburg K (2010). The maternal and placental origins of chronic disease. The placenta and human developmental programming.

[26] Kajantie E, Thornburg K, Eriksson JG, Osmond C, Barker DJP (2010). In preeclampsia, the placenta grows slowly along its minor axis. Int J Dev Biol.

[27] Martyn CN, Barker DJP, Osmond C (1996). Mothers pelvic size, fetal growth and death from stroke in men. Lancet.

[28] McCrabb GJ, Egan AR, Hosking BJ (1992). Maternal undernutrition during mid-pregnancy in sheep; variable effects on placental growth. J Agric Sci.

